# Microarray test results should not be compensated for multiplicity of gene contents

**DOI:** 10.1186/1752-0509-5-S2-S6

**Published:** 2011-12-14

**Authors:** Tomokazu Konishi

**Affiliations:** 1 Faculty of Bioresource Sciences, Akita Prefectural University, Akita 010-0195, Japan

## Abstract

**Background:**

Microarray technology has enabled the measurement of comprehensive transcriptomic information. However, each data entry may reflect trivial individual differences among samples and also contain technical noise. Therefore, the certainty of each observed difference should be confirmed at earlier steps of the analyses, and statistical tests are frequently used for this purpose. Since microarrays analyze a huge number of genes simultaneously, concerns of multiplicity, i.e. the family wise error rate (FWER) and false discovery rate (FDR), have been raised in testing the data. To deal with these concerns, several compensation methodologies have been proposed, making the tests very conservative to the extent that arbitrary tuning of the threshold has been introduced to relax the conditions. Unexpectedly, however, the appropriateness of the test methodologies, the concerns of multiplicity, and the compensation methodologies have not been sufficiently confirmed.

**Results:**

The appropriateness was checked by means of coincidence between the methodologies' premises and the statistical characteristics of data found in two typical microarray platforms. As expected, normality was observed in within-group data differences, supporting application of t-test and F-test statistics. However, genes displayed their own tendencies in the magnitude of variations, and the distributions of p-values were rather complex. These characteristics are inconsistent with premises underlying the compensation methodologies, which assume that most of the null hypotheses are true. The evidence also raised concerns about multiplicity. In transcriptomic studies, FWER should not be critical, as analyses at higher levels would not be influenced by a few false positives. Additionally, the concerns for FDR are not suitable for the sharp null hypotheses on expression levels.

**Conclusions:**

Therefore, although compensation methods have been recommended to deal with the problem of multiplicity, the compensations are actually inappropriate for transcriptome analyses. Compensations are not only unnecessary, but will increase the occurrence of false negative errors, and arbitrary adjustment of the threshold damages the objectivity of the tests. Rather, the results of parametric tests should be evaluated directly.

## Background

Microarray technology has enabled the acquisition of comprehensive quantitative information about mRNA, the transcriptome, in a tissue sample. Because the functions of a cell are primarily determined by expression of the genome, we can assess the state of a cell by examining its transcriptome. However, microarray data may contain irrelevant individual differences as well as noise arising from artifacts of measurement. Indeed, the quality of data generated by microarray assays has been questioned [1,2]. In our efforts to identify essential transcriptomic differences, the significance of observed changes should be evaluated objectively by statistical tests. By the tests, uncertain information can be omitted from further investigations, such as clustering, principal component analysis or pathway analyses.

The test methodologies should be consistent with the data characteristics and the purpose of the test. As with other statistical methods, the principle of a test methodology is based on some assumptions; for accurate analyses, the assumptions should be consistent with the characteristics of the data and the consistency should be checked. Additionally, application of the methodology should be adequate for the purpose of the test [3]. Since a statistical test is in balance between false positive and negative errors, those with overly stringent conditions will produce unnecessary false negatives. Therefore, such strictness is irrelevant when one considers the intrinsic advantage of having complete transcriptome-wide coverage for the discovery of novel findings.

For the tests of gene expression levels, parametric methods such as Student’s t-test or analysis of variance (ANOVA) are frequently used. Generally, these methodologies estimate a p-value, which is the probability that a difference larger than that observed would occur by chance, when actually no difference among populations exists. If the p-value is less than a predetermined threshold, then the observed difference is considered to be significant. Both in t-test and ANOVA, the p-value is calculated by assuming that within group differences are normally distributed; if this assumption does not hold, we cannot accurately evaluate the observed differences among the groups.

Microarray methodology simultaneously measures the expression levels of a large number of genes, and the expression levels of several genes are frequently analyzed collectively. Accordingly, some concerns related to multiple tests [4-6] have been expressed, such as an increase in the family-wise error rate (FWER) [7] or a false discovery rate (FDR) [8,9]. Efforts to control the multiplicity effect are becoming common in microarray studies; according to the assessment of statistical methodologies for microarray analyses conducted by Jafari and Azuaje [10], 10.7 and 18.4% have been applied in research and methodology studies, respectively. Since many tutorial reviews have strongly recommended control of the multiplicity effect [10-14], the proportion may be even higher. Related to this concern, reducing the size of data by focusing to particular genes were also attempted [15,16].

Multiplicity of tests can increase FWER when we group a set of tests together as a family [4-6]; in the presented cases of microarray, the whole set of data from a sample is recognized to form a family. Inevitably, FWER, the expectation of having one or more false positives among the whole family, will become much greater than the expectation of the occurrence of a false positive in an individual test. Therefore, if we wish to control FWER, a compensation of each estimated p-value or threshold is required. A simple solution for the compensation is to use the Bonferroni correction, which compensates for the threshold by dividing it by the multiplicity; i.e., the number of gene contents of a chip. However, since the number of genes in a typical dataset is large, a correction involving division by such a large value will make the test extremely strict. Holm’s procedure [17] obviates this strictness to some extent by assigning different thresholds according to a ranking of p-values. Nonetheless, such methods are considered to be strict since the families of microarray data contain very large numbers of genes.

As the number of tested subjects increases, FDR, the number of false positives among the declared positives, may also increase when large numbers of true null hypotheses are expected [18,19]. On the assumption that all null hypotheses are true, methodology that deals with FDR employs the likely calculation of Holm’s procedure with more relaxed conditions for the compensating threshold; however, the FDR methodology is still stricter than the original tests without compensations.

Despite these efforts to find a practical solution, the methodologies would inevitably make the tests very conservative, increase the false negatives, and reduce the overall information obtained. To deal with the strictness and to regain some of information that may be lost, extremely relaxed thresholds of the tests (10-20%) were recommended [14]. Actually, such relaxed conditions have been used in many studies, and it is not difficult to imagine that the thresholds were invoked ad hoc after the calculations had been performed. Indeed, posterior tuning of the threshold to obtain better achievement was even attempted [20]. Additionally, several offshoots have been produced for FDR methodologies, providing new options to analysts [9,19]. Such alterations to the application would inevitably change the meaning of the methodology and thus, it seems that FDR has been used as an indicator in an arbitrary fashion.

Both FWER and FDR assume high prior probabilities to the null hypotheses; i.e., the population means are identical. In addition, in a recently published book that featured microarray data [21], Efron insisted that Pr(H_0_) is high in large-scale inferences, because most of the cases have small, uninteresting, but non-zero differences. This argument may sound useful for gene selection; indeed, his purpose was to "reduce a vast collection of possibilities to a much smaller set of scientifically interesting prospects". However, this is not necessarily consistent with the current demands of microarray data analyses; since many genes have functional relationships, significance can be tested on such cell functions as well. Interesting functions can be easily found and tested by pathway analysis using databases [15] and/or annotation key words [22]. Rather, if the high Pr(H_0_) scenario unnecessarily increases false negatives, it could limit important information that could be used at higher levels of analyses. Moreover, to negate these small differences, renovation of the null hypothesis and test statistics are required. Nevertheless, Efron did not give any alternative methods, and the complex concept of "interesting" therefore introduced ambiguity in the application of the test. Regardless, in both principle and application, evidence for estimation of Pr(H_0_) is critically important.

We note a trend in the transition of proposed methodologies and the applications described above in that the tightened conditions to deal with the proposed multiplicity have been relaxed enough to employ the unusual handling of the threshold. While it is true that such relaxed application of the test can reduce the number of false negatives, the arbitrariness in choosing both the methodologies and the threshold can damage the objectivity of a test. Indeed, as the transition proceeded, the appropriateness of any of the premises in the methodologies was not confirmed. Additionally, the suitability of the methodologies to the purpose of the test has been left unexamined. For example, no concrete reason has been proposed to explain why the multiplicity should be considered. As will be discussed below, handling of plural test results simultaneously is not a sufficient reason for compensations of the multiplicity [23]. Accordingly, the theoretical bases of present methodologies are rather fragile. In this article, we verify some of the premises against real microarray data from two popular platforms, and we will discuss the appropriateness for the awareness concerning multiplicity.

## Methods

### Data sources

Several sets of Agilent 44K chip data [24] and Affymetrix GeneChip data [25] were obtained from the Gene Expression Omnibus (GEO) repository [26]; the series ID of the data were GSE6089 and GSE3889, respectively (for a complete list, see Additional File 1: List of data ID used in the figures). Mouse liver transcriptome data was obtained from mice administered different diets and the number of measurements in each group was five. Data were normalized by sample according to the three-parameter lognormal distribution model [22] by using SuperNORM data processing service (Skylight-Biotech Inc., Japan); the normalized data are available in the GEO repository under the series ID of GSE25410. Only those data in which signal intensity coincided with the theoretical data distribution were subjected to further analysis.

### Data analysis

Statistical significances in gene expression levels between groups were estimated by using the t-test with Welch’s approximation on normalized gene data. Those were also estimated by two-way ANOVA on normalized perfect match (PM) data of Affymetrix GeneChips, under the assumption that differences in PM data were the sum of group effects and probe sensitivity [22]. The compensations were performed by using p.adjust(stats) function of the R. The threshold used was 0.01 in a two sided manner.

The integrated distribution of gene-wise data variations were compared against the normal distribution using quantile-quantile (QQ) plots. For each gene of the high calorie-fed group, normalized data - normalized within each chip - was collected (n=5). Agilent platform data were selected because an artifact could produce a normal distribution if the average of many PM cell data produced on the Affymetrix GeneChip platform were used, according to the central limit theorem. The collected data were further z-normalized using their mean and standard deviation (SD) to cancel the differences in expression levels and SDs among genes. The renormalized data were then ranked from 1 to 5 according to the signal intensity among the repeats in each gene. In each of the ranks, distribution of the renormalized data was presented at the corresponding theoretical quantiles by using boxplots. The boxes and bars represent the quartiles, and whiskers represent extreme data points that are no more than 1.5 times the interquartile range from the box.

Within-group SD values among the Agilent chip data were estimated by using normalized z-scores. Within-group SD values among Affymetrix GeneChips, which measure a transcript using multiple PM probes, was estimated as the root mean square of the SDs for the probes. The degrees of correlations between the SDs were estimated in Spearman’s ρ by using cor(stats) function of the R.

### Data simulation

A virtual dataset was produced for simulating a scenario in which genes share a common level of noise. The virtual dataset was used to estimate within-group standard deviations and p-values. Each imaginary level was generated by summing the group effect, probe sensitivity, and noise component; these components were produced by generating normally distributed random numbers, of which SDs were set to be identical to the root mean square of the SDs observed in each of the genes of real data. Scripts for the R is available as the Additional File 2.

## Results

### Variation in biological replications obeys normal distribution

**Figure 1:**
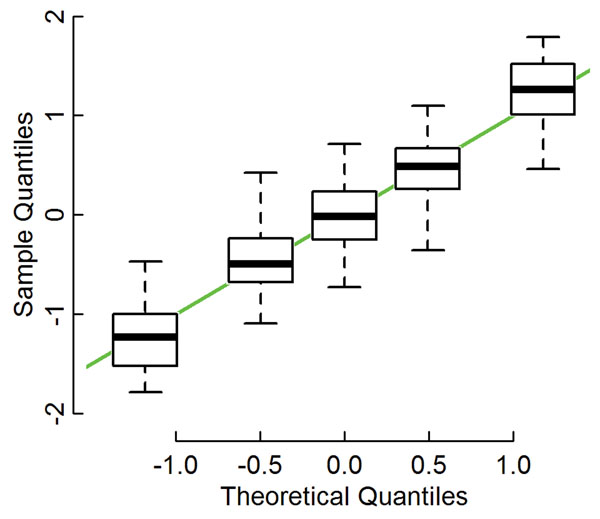
**The general trend of data variations found in an experimental group of mice fed a standard diet.** The data [24] that had originally been normalized in a sample-wise manner were further z-normalized for each gene, and ranked from 1 to 5 according to their intensities in repeated experiments. Distributions of each rank of the double-normalized data are presented using a box and whisker plot for corresponding theoretical quantiles of the normal distribution. The green line shows *y*=*x*.

The inconvenience of using parametric methods is that their premise assumes a certain distribution of the population, i.e., in cases of t-tests and ANOVAs, data variation should be normally distributed. However, it is possible to confirm the actual distribution of data when considering the potential suitability of methodologies. A gene-wise distribution of variation can be verified by comparing the quantiles of real data with their corresponding theoretical values on a quantile-quantile (QQ) plot (Figure 1). Unfortunately, because the number of experimental replicates is limited, assessment of the validity of this relationship for each gene is not very precise. Additionally, this attempt will produce a number of QQ plots equal to the gene contents, and thus the problem of making assessments using numerous vague results becomes apparent.

The general trend of these distributions will be revealed by integrating the gene-wise QQ plots. The integration was performed using expression data further normalized among individual genes, and then determining the distributions of the renormalized expression data for each rank among individual genes (see Methods). The data distribution for each of the ranks was presented using a box and whisker plot and compared with the theoretical value of normal distribution (Figure 1). The median of each rank distributed along the *y*=*x* line, and the height of each box and the length of each whisker showed similar levels of data fluctuations among ranks. Both the coincidence with the normal distribution and the similarity in data fluctuations suggest that the variation of gene expression levels tended toward a normal distribution (Figure 1).

### The compensating method and the number of declared positive genes

To determine the effects of the FWER and FDR compensating methodologies, the test results were compensated accordingly, and the numbers of significant genes were compared (Table 1). The first category of groups compared high calorie and normal diets, with and without Resveratrol administration. The Agilent chips, which measure a gene by using a probe of a single spot, were used in this category. Repeats of five or four measurements were normalized and processed by t-tests. The second category compared the effects of very low fat and normal diets in the *Scd*-/- mouse and the +/+ mouse. The Affymetrix GeneChips, which measure a gene by using several probes separately placed in the chip, were used in this category. The differences between the experimental groups were tested by using two-way ANOVA on the normalized PM data (Methods). Additionally, as the third category of groups, t-tests were performed by using gene expression data, which are estimated by summarizing the corresponding PM data of a gene. Since ANOVA on the PM data can handle a ten-fold larger number of data points, the estimated p-values could become quite low. Therefore, almost half of the genes remained positive in FWER compensations (Table 1, Bonferroni and Holm entries). However, in those values estimated from gene data by t-tests, the compensations severely reduced the number of positive genes, even by FDR compensation, and no differences could be found in some combinations.

**Table 1:** Numbers of positive genes found under the indicated conditions

	Agilent [24]		Affymetrix [25]		PM data [25]	
	-Resv.	+Resv.	*Scd*-/-	*Scd*+/+	*Scd*-/-	*Scd*+/+

parametric	2,104	1,969	3,338	93	10,061	1,035
Bonferroni	16	5	11	0	4,869	179
Holm	16	5	11	0	4,897	179
FDR	230	136	334	0	8,680	370

### Each gene exhibits a unique tendency in stability of expression levels

To select the proper methodology of testing, the noise level of the microarray technique must be known. If data variations are primarily attributed to technical noise, a constant level of noise can be expected among the genes, although the variations observed for each gene will either be over- or underestimated simply by chance. Consequently, a test can be recognized as a part of the repetitions performed under the same conditions, coinciding with Neyman's perspective [27,28], and therefore the observed p-values would fluctuate mainly due to the noise; in such a case, Pr(H_0_) must be high. This could be a valid reason to group a family from the whole set of a sample. Conversely, if the microarray assay is sufficiently accurate and shows individual differences between samples, then each gene will exhibit unique tendencies with respect to the stability of expression levels. If this scenario is true, a correlation in the gene-wise variation of different groups will be apparent. In this case, p-values will show some evidence of variation, and grouping of the family would be unnecessary, negating the FWER scenario.

**Figure 2:**
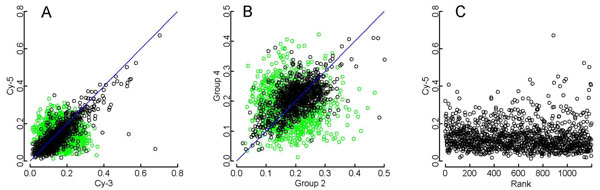
**Characteristics of within-group SDs found in each gene of mice fed with different diets.** A: Between group comparisons in Agilent chip data for standard (Cy-5) and high-calorie (Cy-3) diets [24]; B: Affymetrix GeneChip data for the stearoyl-CoA desaturase 1 mutant (Group 2) and wild type (Group 4) [25]. Black: real data; green: virtual data (Methods). C: Dependence of observed SDs to signal intensities. Standard deviations of the Cy-5 channel of panel A, the standard diet group, plotted against the ranks of mean signal intensities in ascending order. Only 1,000 of randomly selected data are presented in each panel to avoid graphical saturations.

Such a correlation can be evaluated using the standard deviation (SD) within experimental groups; because the data variation is normally distributed (Figure 1), the magnitude of data variation could be evaluated using the SD. Thus, a correlation was observed in scatter plots comparing gene-wise SDs obtained from experimental groups of mice (n=5) fed different diets (Figure 2A and 2B, black circles: the Spearman’s rank correlations were ρ=0.7589 and 0.5731 for panels A and B, respectively). For comparative purposes, an artificial dataset (Figure 2A and 2B, green) was generated to demonstrate the case in which technical noise was the primary cause of the observed data variation. Clearly, the real and the virtual datasets are different. In addition, the SDs observed in the real data did not exhibit any relationship with the signal intensity (Figure 2C; the Spearman’s rank correlations were ρ=-0.002). This independence between SDs and signal intensities implies that the observed correlation between SDs is not restricted to any particular range of signals, precluding the possibility that the effect of noise on weaker signals was responsible for the observed correlation.

### Distribution of p-values is complex

**Figure 3:**
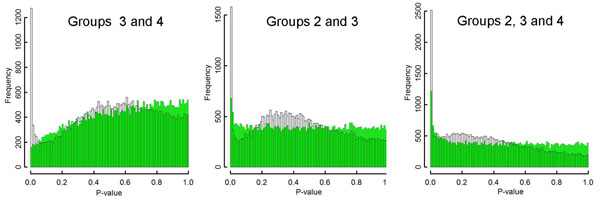
**Frequency distribution histograms for P-values.** The distributions for certain combinations of experimental groups in *Scd*1 mutant studies [25] are shown (open bars). Group 2: *Scd*1 -/-, chow diet; Group 3: *Scd*1 +/+, very low fat diet; Group 4: *Scd*1 +/+, chow diet. The virtual data (green bars) show simulations of cases where most of the variation in data can be attributed to technical noise.

Distribution of estimated p-values will give important information for selecting suitable methodologies for the test, since the origin of data variation can also be estimated from the distribution. If variations in the data can primarily be attributed to technical noise, which is a suitable case for high Pr(H_0_) scenario, then the distribution of p-values can be simulated by using random numbers (Figure 3, green bars). In the simulation, within-group variance, the sensitivity of each PM probe for their target transcripts, and between-group variance were set to be identical to those observed in the real data (Methods). Conversely, if the variation in expression data originates from biological differences and therefore is unique to the genes, then prediction of the p-value frequency distribution will be difficult since it will be affected by the stability of individual genes, which cannot be inferred at present. Figure 3 represents p-value frequencies of real data (open bars), which varied among the combination of groups and are inconsistent with the high-noise scenario described above. The departure of the simulation from the real data suggests that the effect of technical noise on the test results would be limited. Additionally, the rate of true null hypotheses also can be estimated by the distribution of p-values. The case for all true null hypotheses, for example, can be simulated by removing the between-group variance, which will result in a uniform distribution (not shown). In Figure 3, the distribution of the real data is not uniform (open bars); particularly, the smallest p-value class contained considerably more genes than expected from the all null scenario. This outcome shows that the number of true null hypotheses would not be very large.

## Discussion

Variations in the expression levels of each gene within a group were normally distributed (Figure 1), supporting the proposal that parametric tests are appropriate for the analysis of microarray data. Actually, such falsifiability in the principles of a method is necessary to ensure analytical objectivity and it is one of benefits of parametric methods. Even so, the distribution observed in Figure 1 does not necessarily negate the possible occurrence of outliers, such as those attributable to dust during hybridization, and it is possible that such outliers could alter the test results. Rather, the application of robust alternative functions, such as trimmed-mean and median absolute deviation to assess the data distribution parameters of the tests, may be applied to resolve such problems.

The gene-wise tendency observed for within-group SDs (Figure 2) as well as the complex distribution of p-values (Figure 3) revealed that the primary origin of data variability was not due to technical noise, the level of which would be common to all genes. The primary origin of data variation therefore appears to be related to biological differences between individual samples, which could be taken to reflect the variability in the expression of individual genes. While the quality of microarray assays has been questioned [1,2], the actual level of noise is therefore low enough to reflect biological differences among samples; thus, considerable improvements have been made to chips, reagents, and experimental protocols [29], and advances in data analysis have resolved many of the problems previously associated with the normalization of data [22], thereby improving the robustness of the assays and reproducibility of observations.

As has been described before, the main purpose of testing significance of a gene is to reduce uncertain signals in higher level of analyses. Even if the technical noise is low, individual organisms have biological differences, and some genes may frequently and drastically change their expression levels according to biological requirements. To observe between-group differences for such genes, the tested data may lack a sufficient number of biological repeats. Such volatility or stability of a gene can be estimated from within-group differences found in the forms of SDs (Figure 1), and the significance of the between-group differences can be tested by using parametric methods. It should be noted that the test is performed for each gene independently, since both the observed SD and the between-group differences are unique to the gene. In this sense, there is no reason to combine some test results in order to evaluate them.

Therefore, the suitability of the definition of a family by the gene contents of the microarray data should be reconsidered. Actually, although it is a very crucial decision, there are no fixed rules for how we determine a family [5]; rather, a family should be decided according to the purpose of the test [4-6]. In cases in which we wish to select only a few genes among the whole set of data and just concentrate on those genes, FWER could be important because the genes definitely should not be false positives. In the early years of microarray technology, such an application could be possible; however, in practice, the expressional changes are often confirmed by other methods or by a different level of observations such as enzymatic activities, even in such experiments. Additionally, we rather tend to analyze the transcriptome as a whole, identifying trends in global changes. It is true that as the number of items to be analyzed increases, so too does the FWER. However, a few false positives may not be problematic, since transcriptome-wide observations such as primary component analysis or pathway analysis will not be much affected by a single false positive, since we would be handling hundreds of true changes. Consequently, we do not need to control FWER for microarray data analysis, unless the purpose of the tests is very sensitive to an error.

The appropriateness for the concerns of increasing FDR should also be reconsidered. Originally, the concern over FDR was based on the high probability of a true null hypothesis [18,19]. In a test for a true null, the p-value will be given by random effects and hence would not support the evidence; consequently, the expectation of a false positive should be estimated by using the threshold and not the p-value in the premise of FDR methods. However, each subject of transcriptome analysis is a so called 'point null' or 'sharp’ hypothesis, i.e., a double-sided test for coincidence of continuous variates, so rarely could this outcome be true in principle. In particular, we define a population within each gene under a specified set of experimental conditions. The expression level of the gene under those conditions can be represented by the center of the population's distribution, which would be normally distributed (Figure 1). The null hypothesis of each test is that the centers of the compared populations are identical. Since expression levels are continuous values, the probability of the center having any particular value is null, and the probability of coincidence in some populations's centers is also null. Actually, the distribution of the p-values supports the rare occurrence of a true null (Figure 3, open bars). Therefore, as the premise of high probability of true nulls contradicts reality, the concern of increasing FDR is not applicable for transcriptome analyses.

The idea that compensation is unnecessary would also be true with respect to data obtained in sequencing-based methodologies, such as RNA-seq [30], when a transcript is measured with a sufficient number of reads. Although those data are intrinsically discrete, they can be viewed as continuous data in a practical sense with a large number of reads. However, the precision of the data will become worse with fewer reads. The expected precision can be estimated according to the binominal distribution model; for example, reads of 100 and 10 out of one million reads would have a 95% interval estimate of 81-121 and 4.8-18.4, respectively. Such technical noise will be added to the individual differences; in extreme conditions, the random effects will practically determine the test results. Under such conditions, we should address the multiplicity problem. Since Pr(H_0_) would not be uniformly high but a function of the numbers of reads, the FDR [30] would be too conservative; further investigations will be required for more suitable compensation.

We should not compensate for multiplicity of tests unless there is a good reason for doing so. It is now obvious that the high Pr(H_0_) scenario is against the evidence presented here. This means that the currently proposed problems for multiplicity in microarray data, FWER [7] and FDR [8,9], have been negated in their principles. Additionally, the excessively strict conditions will increase false negatives (Table 1) and thereby disturb the higher levels of analyses. Indeed, judgment of whether a finding is interesting or not is not necessarily performed for each gene; rather, it is important to remove "uncertainties about the direction" cases [3], in which we cannot distinguish "up" or "down" expressional changes from the following analyses.

A far more important problem should concern the design and management of experiments. As was discussed, the principal source of noise is in individual differences among samples, but not in the measuring technique. Since experiments are performed by using a limited number of replicated experiments, any small differences arising in experimental conditions among groups can introduce significant biases that may manifest as a global level of false positives. Unfortunately, such experiment-based false positives cannot be controlled by any of statistical methods in principle, since what was observed actually occurred in that experiment. To control for such biases, experimental groups should be randomized (e.g., placement of cages or pots in experiments) beyond groups, to avoid being treated in any specific order.

## Conclusions

Microarray analysis is accurate enough to observe individual differences among samples, and performing parametric tests for the results is recommended to confirm the significance of transcriptomic differences among groups. It should be noted that, in most of the cases, FWER or FDR should not be considered with respect to the tests; these procedures are inappropriate for global transcriptome analyses and will increase false negative errors, eliminating information that would otherwise be obtained. Rather, strict control for false positive errors should be considered in higher levels of analyses, but not in the gene-wise case. A more important source of problems would be in the design and management of the experiment, since any biological differences of conditions among groups will produce false biases in the data.

## Competing interests

The author declares that he has no competing interests.

## Supplementary Material

Additional File 1 **List of data ID used in the figures.** The list of GEO ID of the data used in the calculations. Click here for file

Additional File 2 **Scripts for the R.** Scripts used to perform 2 way ANOVA and the simulations. Click here for file
