# Supplementary material for: Microarray test results should not be compensated for multiplicity of gene contents
Source: BMC Syst Biol. 2011 Dec 14;5(Suppl 2):S6. doi: 10.1186/1752-0509-5-S2-S6 (PMC3287486; doi:10.1186/1752-0509-5-S2-S6)
Supplement: Additional File 1 — List of data ID used in the figures. The list of GEO ID of the data used in the calculations. [file 1752-0509-5-S2-S6-S1.doc]

| Fig. 1 | Normal Diet, Ad Lib, Liver | Cy-3 | GSM140954 | GSM140955 | GSM140956 | GSM140957 | GSM140958 |
| --- | --- | --- | --- | --- | --- | --- | --- |
|  |  |  |  |  |  |  |  |
| Fig.2 A  Table1 | C57BL/6NIA Mice, 18months of age,  High Calorie, Ad Lib, Liver | Cy-3 | GSM140959 | GSM140960 | GSM140961 | GSM140962 | GSM140963 |
| Fig. 2AC  Table1 | C57BL/6NIA Mice, 18months of age,  Standard Diet, Ad Lib, Liver pools | Cy-5 | GSM140959 | GSM140960 | GSM140961 | GSM140962 | GSM140963 |
|  |  |  |  |  |  |  |  |
| Fig. 2B  Fig 3AC  Table1 | Scd1+/+ on chow diet |  | GSM88887 | GSM88888 | GSM88889 | GSM88890 | GSM88891 |
| Fig. 2B  Fig 3BC  Table1 | Scd1-/- on chow diet |  | GSM88877 | GSM88878 | GSM88879 | GSM88880 | GSM88881 |
| Fig 3ABC  Table1 | Scd1+/+ on very low fat diet  (TD03045 from Harlan Teklad) |  | GSM88882 | GSM88883 | GSM88884 | GSM88885 | GSM88886 |

Table 1: List of data ID used in the Figures.
